# Social network analysis and agent-based modeling in social epidemiology

**DOI:** 10.1186/1742-5573-9-1

**Published:** 2012-02-01

**Authors:** Abdulrahman M El-Sayed, Peter Scarborough, Lars Seemann, Sandro Galea

**Affiliations:** 1 Department of Public Health, University of Oxford, Oxford, UK; 2 Department of Epidemiology, Columbia University, New York, NY, USA; 3 College of Physicians and Surgeons, Columbia University, New York, NY, USA; 4 Department of Physics, University of Houston, Houston, TX, USA

**Keywords:** Complex systems, epidemiology, social factors, social class, ethnicity, modeling, networks

## Abstract

The past five years have seen a growth in the interest in systems approaches in epidemiologic research. These approaches may be particularly appropriate for social epidemiology. Social network analysis and agent-based models (ABMs) are two approaches that have been used in the epidemiologic literature. Social network analysis involves the characterization of social networks to yield inference about how network structures may influence risk exposures among those in the network. ABMs can promote population-level inference from explicitly programmed, micro-level rules in simulated populations over time and space. In this paper, we discuss the implementation of these models in social epidemiologic research, highlighting the strengths and weaknesses of each approach. Network analysis may be ideal for understanding social contagion, as well as the influences of social interaction on population health. However, network analysis requires network data, which may sacrifice generalizability, and causal inference from current network analytic methods is limited. ABMs are uniquely suited for the assessment of health determinants at multiple levels of influence that may couple with social interaction to produce population health. ABMs allow for the exploration of feedback and reciprocity between exposures and outcomes in the etiology of complex diseases. They may also provide the opportunity for counterfactual simulation. However, appropriate implementation of ABMs requires a balance between mechanistic rigor and model parsimony, and the precision of output from complex models is limited. Social network and agent-based approaches are promising in social epidemiology, but continued development of each approach is needed.

## Introduction

### Social epidemiology and systems thinking

Social epidemiology is concerned with the social variation in, and the social determinants of the distribution of health and disease [1]. This branch of epidemiology is fundamentally interested in the influences of social factors--such as individual attributes (i.e., social class and ethnicity) [2,3]; behaviors (i.e., diet and physical activity) [4,5]; constructs of social interaction (i.e., social support and social cohesion) [6]; contextual influences (i.e., neighborhoods and regions) [5,7]; and the influences of the allocation of individuals in space (i.e. race/income segregation) on the distribution of health and disease in populations [8,9]. By patterning exposure to disease risk factors, social factors themselves become fundamental determinants of health [10]. Social epidemiology has made great strides during the past two decades. However, as the field grows, it is becoming readily apparent that some of the its tools may be limiting. In particular, the reductionist linear models that are the *lingua franca *of epidemiologic analyses are limiting in important ways.

First, the dynamics of populations, in terms of health and disease, emerge from the behaviors and interactions of the heterogeneous individuals that comprise them. In this way, interaction undergirds many of the mechanisms that mediate the social production of health and disease. These interactions may operate both on the macro-scale between social exposures acting at multiple levels, and on the micro-scale between individuals within populations. Interactions challenge the current epidemiologic toolkit in several ways. As social factors may interact in complex ways to determine health and disease risk, the current "risk factor" approach to epidemiology, which emphasizes decontextualized, independent effect measures for exposures may not be appropriate [11,12]. For example, studies have demonstrated that the relation between ethnicity and health indicators may be modified by ethnic density in the area of residence; this observation has been documented for health indicators including adverse birth outcomes [13,14], asthma [15], psychopathology [16], suicide [17-19], and mortality [20]. This observation challenges our current approaches because it suggests that the relation between ethnicity and health may be heterogeneous, and that conceptualizing this relationship independently of social context may be flawed. Furthermore, social variability in health may be mediated by the degree and nature of social interaction within and between social groups. In this regard, several studies have shown that social interaction may transmit non-infectious disease outcomes [21-24]. Furthermore, research about the health influences of social interactions suggests that population-level modes of social interaction, such as social cohesion, social capital, and social support, may shape population health and disease distribution [6,25,26]. Ultimately, however, social interaction does not lend itself to the reductionist analytic paradigm that we employ, as potentially important social interactions between individuals in a population violate the central assumption of independence of observations in regression approaches.

Second, population dynamics feature nonlinearity, whereby change in disease risk is not always proportional to the change in exposure, and feedback, where disease can modulate exposure just as exposure can modulate disease. These dynamics are not often explored in social epidemiology, although they may have profound implications for population health. For instance, a central observation in social epidemiology is that low social status predicts poor health [27]. However, poor health can also predict low social status [28]. Therefore, mutually reinforcing in a positive feedback loop, low social status and poor health may ultimately converge, with reinforcing implications for a third social ill--inequality (which itself plausibly feeds back on low social status and poor health) [29,30]. As is characteristic of positive feedback loops, the relationships between social status, health, and inequality are likely to feature nonlinear, accelerating behavior because of amplification at each turn of the loop. As an illustration of the inability of the current epidemiologic paradigm and toolset to negotiate these dynamics, consider the use of directed acyclic graphs (DAGs) in traditional epidemiologic analyses. DAGs are mental models used to specify and formalize the causal relationships between exposures and outcomes. However, like the regression models they educate, these mental models, by definition, forbid cyclical relationships between exposure and outcome, and therefore the feedback and reciprocity that likely characterize the true relationships between them.

Third, the counterfactual conceptual framework that underpins epidemiologic inquiry falls short when considering both fundamental social causes and macrosocial causes of disease. Our etiologic understanding of the social determinants of disease rests on the counterfactual exercise of contrasting outcome occurrence probabilities corresponding to two or more mutually-exclusive exposures [11,12]. However, social factors, of fundamental importance in social epidemiology, such as race, ethnicity and gender, are *attributes *of individuals, rather than exposures. Because these attributes are fundamental to identity, authors have argued that the counterfactual approach is theoretically implausible [30-33]. Similarly, understanding macrosocial causes requires the assumption that a counterfactual universe could be unchanged barring a large-scale social cause. However, causes across levels are inevitably interlinked, suggesting that an alternate universe comparable to the present universe, save changes in a macrosocial influence, may also be implausible.

These three challenges may be limiting the progress of social epidemiology at this stage in its evolution [34], and have resulted in calls to adopt newer methods that can overcome them [32,34-36]. Several authors have suggested the adoption of systems approaches in social epidemiology as a way past these challenges [37-39]. "Systems thinking" suggests that complex dynamic systems, such as populations, which feature multiple interdependent components whose interactions may include feedback, non-linearity, and lack of centralized control [40], are best understood holistically [41]. This epistemological approach is best contrasted to "reductionism", which suggests that systems are best understood by aggregating information gathered via the independent study of their components. By contrast, a systems approach implies that the dynamics and behavior of a system are different, qualitatively, from those of the sum of its parts. A systems approach, therefore, emphasizes the dynamics of *relationships *between components of a system, rather than the *characteristics *of those components themselves [41,42].

Two systems approaches that may be particularly useful in social epidemiology include social network analysis and agent-based modeling. With respect to social epidemiology, the first involves the characterization of the structures of social networks or subsets of these networks to understand their influence on health behaviors and outcomes. The second involves the use of stochastic computer simulations of simulated individuals, in simulated space, over simulated time to understand how macro-level health and disease distribution patterns may emerge from explicitly programmed, micro-level health behaviors, social interactions, and movement of these individuals in their environments.

A developing body of work has begun to apply these approaches to address social epidemiologic research questions. For example investigators have used these approaches to better understand the social etiology of complex conditions, [21-24,37,43-46] such as obesity [24,43]. Particularly compelling is a recent high-profile study by Christakis and Fowler [24], which used social network analysis to demonstrate the spread of obesity via social relationships in a social network. Another study used stochastic networks nested within agent-based models (ABMs) to assess strategies for population-level obesity prevention [43]. Two more recent studies used agent-based models to understand the mechanisms underlying socioeconomic disparities in diet quality [47], and to assess how resource allocation may influence socioeconomic disparities in walking behavior [48].

While these papers are good early examples of the adoption of complex systems approaches to social epidemiologic inquiry, the field remains young. Here, we aim to synthesize the extant literature that has called for applications of these approaches in population health. We will begin with an examination of each method and its approach, and then examine each method's strengths and applicability with regard to social epidemiologic research, suggesting particular avenues where each may be appropriate. Finally, we will discuss limitations to the application of each method in the field.

## Analysis

### Social network analysis

#### A summary of social network analytic approaches

In the context of social epidemiology, social network analysis involves the characterization of the structures of social networks or subsets of networks so as to understand the flow of health-relevant factors (i.e., disease, information, social support, etc.) between network nodes. Network approaches emphasize the structural characteristics of networks rather than the characteristics of their nodes--this implies that the social ties that bind actors have important consequences for their behavior [49]. Social network analysis is particularly interested in the patterns and implications of relationships between social actors [50], and is therefore most valuable in characterizing population-level outcomes when there are relational characteristics involved in the behavior of networked individuals [51].

Social network analysis has three main branches: (a) network visualization and (b) network characterization, each principally descriptive, and (c) emerging methods around stochastic and longitudinal network analysis [50-52]. Network visualization is the process of diagramming network connections in two and three-dimensional space so as to visualize network structure and relationships [51]. Network characterization involves analyses directed toward understanding the roles played by individual actors, subgroups of actors, or overall network structures in characterizing the flow of factors of interest within networks. For example, at the level of the individual, analyses might address the number of connections a particular actor has within a network, the degree to which the actor bridges between other actors in the network, the social distance (measured in relationships) between an actor and other actors, or the degree of connectedness of an actor relative to others [50-52]. By contrast, analyses of network substructures or full networks may address degrees of connectivity between actors, the degree of centralization or hierarchy in a network, or lengths of paths between particular actors of interest [50-52].

A third wing, emerging from network characterization, aims to develop methods for inferential analysis and hypothesis testing regarding network influences. These methods, still in development, include stochastic, and longitudinal network analytic techniques [51,52]. Longitudinal network analysis allows investigators to study temporal changes in networks, their characteristics and dynamics, and/or the characteristics of their constituent parts, while stochastic analytic techniques allow for the construction of network models for use in simulations [52].

#### Applicability of social network approaches in social epidemiologic research

Social epidemiology is interested in the influences of social factors on health and disease distribution in populations. Because of its focus on social interaction as a potential driver of individual and collective characteristics and behaviors, social network analysis has the potential to yield valuable insight about the social production of health and disease in three areas: understanding the social contagion of non-infectious exposures and outcomes [21-24,44-46], understanding the role of social network structure as a determinant of population health disparities, and understanding the influences of modes of social interaction, such as social support, social cohesion, and social capital on population health [51].

#### 1. Understanding the social contagion of non-infectious exposures and outcomes

Social network analysis is particularly useful for studying how social phenomena spread through social networks and influence health in this manner. Using social network analysis, several studies have demonstrated the spread of non-infectious conditions through social networks, including obesity [24], smoking [20,44], alcohol use [45], back pain [46], teen substance use [21,22], and general well-being [23]. These findings suggest that other complex exposures, conditions, and diseases of population health interest also may be, in part, communicable.

However, the current literature that has employed social network approaches to understand the communicability of non-infectious health outcomes has only scratched the surface of the applicability of these approaches. For example, while it has been suggested that obesity may be communicable via network ties [24], little is known about heterogeneity therein--why might some contacts of obese individuals become obese while others may not? Moreover, does the answer to the previous question lie in characteristics of the obese contacts, the characteristics of those exposed, or in characteristics of the relationships they share? Finally, while these approaches have yielded insight about the social etiology of these outcomes, we know little about the potential for interventions to exploit network influences.

#### 2. Assessing the role of social network structure as a determinant of population health disparities

Differences in the density and character of social networks connecting individuals within different social strata could partially explain population-level disparities between those strata. In that sense, these approaches may be used to study the mediation of health disparities by characteristics of social networks. For example, ethnic minority groups in high-income contexts have higher risk for obesity than their white counterparts [53]. Moreover, social networks have been shown to be ethnically and racially segregated, and those among ethnic and racial minority groups have been shown to be stronger and more highly-interconnected than those among their White counterparts [54]. If obesity has a communicable etiology, as findings from Christakis and Fowler suggest [24], then it is plausible that obesity may spread faster and more completely in more dense social networks, and therefore faster and more completely among minority groups than among whites. In this manner, differences in social network structure among ethnic majority and minority groups may influence the spread of obesity among them, helping to explain disparities in obesity. Therefore, differences in social network characteristics across strata at several levels, including ethnicity, socioeconomic status, and education at the individual-level, as well as deprivation or ethnic density at the area-level, may mediate differences in health between these groups. Network approaches, used in this way, may yield insight into the mechanisms underpinning population health disparities.

#### 3. Social network analysis and the influences of social interactional constructs on population health

With the potential to distinguish between the effects of the nature and volume of social interactions, the environmental contexts in which they exist, and the characteristics of the actors involved, social network approaches provide the correct *framework *within which to conceptualize and operationalize modes of social interaction, like social support and social capital [51], which have been shown to influence population health [6,55,56].

This is an important methodological development, as standard regression-based approaches are not suited to analyze these constructs because they are fundamentally relational, and therefore violate the assumption of independence of observations. At best, some investigators have attempted to use multilevel regression techniques to incorporate a measure of social support, cohesion or capital at the area-level [57,58]. Aside from the issue of confounding by other area-level factors correlated with these interaction constructs, these approaches cannot accurately capture the role of these exposures in heterogeneous populations, where social support is not evenly distributed. Rather, because social network analysis allows investigators to more accurately represent and analyze these constructs, this methodology is well situated for studies of the relationship between social interaction and population health.

#### Limitations to the application of social network approaches in social epidemiology

Social network analysis is not without limitations. Two principal limitations are the implicit trade-off between the use of network analytic techniques and generalizability in network data, and the problem of confounding by either homophily and/or by shared environments in studies about social induction of exposures or outcomes through networks.

With regard to the first limitation, beyond data about the characteristics of individuals in networks (traditional data collected in health surveys), social network analysis requires data about the relationships between individuals. Traditional sampling techniques designed to improve study generalizability by randomly sampling across environments are not conducive to using network approaches, as the data yielded about relationships from these techniques is not of sufficient completeness or quality to support them. For example, a national survey may collect data about exposures and health outcomes among a random sample of the population, as well as about the number of social contacts each respondent has, but it does not collect data about exposures and outcomes among those respondents. Conversely, studies attempting to maximize the quality of network data may have limited generalizability. Therefore, because of cost and feasibility constraints, investigators interested in applying network methodologies in their work may be forced to balance tradeoffs between the analytic benefits of social network approaches and the importance of generalizability when planning epidemiologic studies.

Studies about the networked spread of exposures or disease are primarily interested in social induction, or the causal influence of social interaction on behavior or health outcomes. The second limitation to the use of social network analysis in social epidemiologic research is the difficulty of adjusting for either homophily or environmental effects, two potential confounders in analyses about the causal influence of induction [59-61]. Homophily is the tendency for agents with similar *a priori *likelihoods of developing an outcome to preferentially form social relationships [24,54]. Along with homophily, shared spatial environments between individuals in networks can also confound studies of social induction. A series of recent studies have drawn attention to the difficulty of disentangling social induction from confounding by homophily and environmental effects (see work by Cohen-Cole and colleagues [60,61]). Given these challenges, the development of methods to differentiate induction from its potential confounders remains an active area of research [59].

### Agent-based models

#### A summary of agent-based modeling

ABMs are stochastic computer simulations of simulated "agents", or individuals, in simulated space, over simulated time. These models allow macro-level behavioral patterns to emerge from explicitly described, micro-level behaviors, interactions, and movements of agents in their environments. Because model conceptualization and parameterization take place "from the bottom, up" these models are ideal for assessing "emergence," or macro-level patterns that arise from micro-level behavior [62]. Emergence, an idea that is central in systems sciences, may allow for tremendous new insight into important questions in the social and natural sciences [63].

Agent-based approaches are particularly appropriate when: 1) individual agent behavior is complex, featuring learning and adaptation, feedback loops, and/or reciprocity; 2) when heterogeneous environments can influence agent behavior and interaction, and agents are not fixed in space or time, and 3) when inter-agent interactions are complex, non-linear, and influence agent behavior [39,62]. In sum, agent-based approaches are ideal when agent behavior is a complex function of agent attributes and characteristics, environments, and inter-agent interaction over time.

Agent-based modeling requires the investigator to explicitly describe and program agent characteristics and updating rules during implementation. This includes specifying agent characteristics and behaviors, as well as changes to them with time (e.g., learning and adaptation). Agents can be nested within social networks that influence the degree and character of inter-agent interaction, and social interaction can be programmed to influence future behavior. Moreover, investigators can explicitly define the space within which agents are situated through time, as well as the influence of that space on agent behavior with time.

ABMs are particularly well-suited for research that is concerned with understanding social processes because they maintain the centrality of the individual agent and its attributes, characteristics, and behaviors in the production of population-level phenomena. This is contrasted with other methods, such as regression models or differential equations (e.g., laws that determine dynamics of predators and prey), which focus on aggregated data [63]. For this reason, agent-based approaches have become increasingly common throughout the social sciences, with applications in economics [64,65], sociology [66], and political science [67,68].

#### Applications of agent-based modeling in social epidemiologic research

ABMs place a focus on the individual and the individual's characteristics and interactions in time and space. They also allow investigators to run multiple simulations under various model conditions, thereby isolating the effects of particular conditions on outcomes of interest. Therefore, this approach has the potential to move social epidemiology forward in four important ways. First, ABMs move beyond the limitations of reductionist approaches that have centered social epidemiology around measurement of decontextualized "risk factors" that do not account for interrelatedness and reciprocity between social exposures. Second, ABMs may play a useful role in helping us understand causal inference in social epidemiology. Third, this approach may allow investigators to articulate and explore mechanisms that underlie our understanding of the social production of health. Fourth, agent-based approaches may provide a more robust means to forecast the outcome of policy interventions.

#### 1. Moving away from "independent effects" in social epidemiology

Individual attributes and behaviors, social interaction, and environmental factors are largely interdependent, interacting to shape health and disease distribution. However the dominant reductionist models that are used in the field are limited in their ability to analyze interactions, feedback, and reciprocity between exposures with one another and with outcomes [34], For these reasons, several authors have challenged the assumption that elucidating risk factors or "independent effects" should be the object of epidemiology [32,34,36,37].

ABMs present a departure from this approach. They allow investigators an opportunity to model the influences of individual, inter-individual, and environmental factors in a mechanistically cogent manner. Unlike reductionist approaches, ABMs are uniquely suited for the assessment of the simultaneous etiologic effects of heterogeneous population characteristics, social interaction, and the environment. They also allow for the exploration of mechanistic interactions, feedback loops, and reciprocity between exposures and outcomes operating at multiple levels in the etiology of complex conditions. For these reasons, these methods may allow us to move beyond the reductionist measurement of independent "risk factors", to a more realistic and nuanced understanding of the complex production of health and disease.

#### 2. Agent-based models and causal inference in social epidemiology

ABMs may also lend themselves to the question of causal inference in social epidemiology. Social epidemiologists are centrally concerned with understanding the social production of disease [12]. This involves the counterfactual exercise of contrasting outcome occurrence probabilities corresponding to two or more mutually-exclusive exposures [11,12].

In agent-based modeling, investigators program initial conditions and update rules that specify the characteristics of agents, embedded in networks, and placed within spatial contexts, all of which influence update rules that specify stochastically-applied changes in agent behavior and characteristics each time-step, or simulated unit of real time. Assuming no changes in initial conditions and update rules, each simulation is identical within the bounds of stochasticity. Calling this initial simulation the "control", an investigator can then simulate other models where only one aspect of the initial conditions or the update rules is changed in each simulation--these being "experimental" simulations. Because both experimental and control simulations are applied to the same population of agents, this approach may bypass Holland's "Fundamental Problem of Causal Inference", which exists when it is impossible to observe the effects of multiple exposures on the same unit in epidemiologic analysis [31]. Therefore, by comparing any number of "experimental" simulations to the "control" simulation, the investigator can simulate population-level experiments using the ABM [63]. This experimental framework is particular powerful in social epidemiology, where there are considerable logical limitations to causal inference from observational models [11,12,69], and where randomized social interventions are fraught with logistical, ethical, and logical limitations of their own [12].

Therefore, ABMs allow investigators to use agent-based counterfactual simulations (ABCs) in social epidemiologic inquiry. In this way, they may allow for more robust analyses of the effects of counterfactuals that challenge reductionist approaches. These include social factors that range from characteristics as fundamental in social epidemiology as race or gender, to broader social phenomena, such as macrosocial exposures, which are difficult to operationalize using real world data. ABCs allow investigators to identify the etiologic effects of these factors by positing implausible counterfactuals that deductively clarify their influences on population health. For example, an investigator interested in the effects of segregation on inequalities in obesity in a real population could counterfactually simulate the effects of complete desegregation, comparing the degree of inequality in this simulation to that of a segregated simulation. Another, interested in the effects of income inequality on suicide, could simulate the effects of perfect income parity relative to varying levels of income inequality on suicide overall and by subgroup. This method of analysis poses considerable strengths over current analytic paradigms, largely relegated to cross-sectional ecologic analyses of observational data between vastly different societies, limited by comparability and the inability to properly adjust for confounding.

There is another potential benefit of this counterfactual approach in social epidemiology: a more unified conceptualization of disease etiology. Kaufman and colleagues argue that it is most appropriate to articulate exposures of interest as defined interventions that would eliminate them [12]. Conceptually similar to randomized social interventions in their experimental ontology, ABCs also force us to articulate exposures as defined interventions--the programmed difference between the "experimental" simulation and the "control". In this sense, investigators using ABCs must frame exposures of interest in terms of the counterfactual in which the exposure in question does not exist, an exercise that serves to clarify and define that exposure more accurately.

#### 3. Agent-based counterfactual simulations and the exploration of etiologic pathways

The opportunity for experimentation via ABCs allows us to explore mechanisms that underlie the social production of disease. The population health implications of perturbations on a hypothetical control model are driven both by initial conditions, and by mechanisms underlying update rules that relate exposures with one another or with outcomes. ABCs that attempt to replicate empiric observations can be run to clarify the mechanistic relationships necessary in a model to yield real-world outcomes. In this way, ABCs can allow investigators to study mechanisms underlying the production of health and disease.

#### 4. Modeling the effects of social policy interventions on health

A potentially important contribution of ABMs is the opportunity to "test" the outcomes of policy interventions. This represents a key departure from current methods, as traditional regression-based approaches are limited with regard to yielding policy-relevant inference in several ways. First, inference of policy implications from regression models is limited to interpreting the appropriate targets for interventions from "independent" effect measures. This is limiting, as these effect measures have no direct relevance to potential intervention schemes. Second, because of limitations to regression models with regard to the appropriate representation and operationalization of macrosocial phenomena, macrosocial interventions are difficult to study in this manner. Third, because the outcomes of regression models are specific to the data analyzed, policy relevant analyses using these approaches should be performed on data from the population within which a proposed policy intervention is to be enacted, necessitating often costly and time-consuming data collection. Fourth, as discussed above, regression-based approaches are not equipped to account for reciprocity, feedback, or non-linearity in relations between exposures and outcomes, which may be important in understanding the effects of policy interventions on outcomes (intended and unintended).

By contrast, the counterfactual experimental approach may allow for the simulated development and "testing" of proposed health policy interventions [37,39]. Investigators can tailor initial conditions to populations within which a proposed policy intervention is to be enacted. The policy intervention itself can then be operationalized as a counterfactual simulation and proposed interventional strategies can be compared head-to-head or to baseline.

#### Limitations to agent-based approaches in social epidemiology

Agent-based approaches require the investigator to balance the importance of mechanistic rigor (i.e. the inclusion of relevant factors) and model parsimony (i.e. overcomplicating the model) [39,62]. In this sense, the process of model implementation should be tailored to questions of interest to avoid unnecessary complexity [62]. However, model tailoring may be logically problematic, as purposive model tailoring implies the *a priori *exclusion of factors that should have no *apparent *influence on the outcomes of interest. However, a central argument for agent-based approaches is the ability of these models to yield emergent phenomena, which rely on the aggregation of complex micro-level processes to yield macro-level insight. The very notion of emergence suggests that the aggregation of micro-level factors is likely too complex to allow for *a priori *exclusion of any factor that could potentially influence, however indirectly, the outcome of interest--or that it is impossible to know which factors may have influences on outcomes in the first place. Alternatively, because these models are stochastically implemented, added computational factors will generally (although not in all cases) increase the degree of uncertainty in simulation outcomes, imposing a pragmatic limit to the number of factors that can be included in a given model.

Along that line, another limitation is that ABMs, as computer simulated models, produce quantitative output, tempting investigators to quantitatively interpret their findings. However, ABMs can include any number of factors, each parameterized from any number of sources and introducing their own biases and assumptions into model output. Therefore, quantitative interpretation of ABM output may not be appropriate [39,62]. This is particularly true in highly complex simulations drawing upon multiple sources for parameterization. In that sense, ABMs may not be particularly useful when attempting to forecast absolute population health indicators, such as population prevalence or incidence of a given outcome. Rather, these models are better suited for etiologic inquiry reliant upon qualitative comparisons of output between counterfactual simulations. One tool that may allow for more objective assessments of qualitative differences in output is the use of Monte Carlo simulations [70]. Averaging model output over several Monte Carlo simulations can provide confidence intervals on model estimates that can then be used to elicit measures of significance on differences in output between counterfactual simulations.

Another set of limitations arises when considering validation. ABMs, by their nature as systems models, are difficult (and sometimes impossible) to validate completely. Generally, there are two strategies an investigator might use to validate a model by situating it in reality. The investigator can 1) use real data to parameterize the model, or 2) "work backwards" by building a model from conceptual relationships, the findings of which can then be compared to real world observations. In this way, either the relationships between factors in the model or the outcomes of the model can be validated, but rarely both.

Both of these approaches are limited. In the first case, the validity of counterfactual simulations depends on the valid operationalization of factors in the model, which is nearly impossible to verify, even when those factors are operationalized from real data. In the second case, where a model parameterized based on conceptual relationships between factors in the model is then validated using real world observations, there is the threat of affirming the consequent. In this case, while a particular configuration of factors in the model might produce outcomes that predict the observed data, it is possible that there are many other plausible configurations that would also predict the observed data, and no way to affirm that the investigator has isolated the one that operates in reality. However, model building is not idiosyncratic and model construction should be educated by current knowledge about the phenomena in question increasing the likelihood that the specified conceptual model is accurate.

More generally, a central premise underlying the systems approach, as discussed above, is that the "whole" may be different from the sum of its parts. Therefore, it is plausible that outcomes that emerge from systems models, such as ABMs, may contradict findings from reductionist approaches [62,63]. There is a conceptual limitation to validating emergent findings using models that cannot, by definition, capture this phenomenon. Thus, it may be difficult to differentiate new insight yielded by models which capture emergence from spurious findings, because, by definition, another model capable of capturing emergence would be needed to do so.

Another important limitation to the use of agent-based approaches is that they may be beholden to regression-based parameters in their construction. Therefore, they may incorporate into their findings many of the same biases and limitations that arise from reductionist regression-based approaches.

Finally, agent-based approaches can be computationally intensive. Therefore, these approaches can require considerable computing resources for efficient use [62].

## Conclusions

The systems approaches discussed here, social network analysis and agent-based modeling, have the potential to reframe social epidemiology. Chiefly, these tools allow investigators to move social epidemiology beyond the "independent effects" paradigm that some describe as conceptually inappropriate [32,35] and reframe our pursuit of the complex social causes of health and disease in a holistic framework. In addition, these approaches allow investigators to understand the etiologic implications of heterogeneity within the population, social interaction, and environmental influence simultaneously, and to explore mechanistic interactions, feedback loops, and reciprocity between exposures and outcomes. Moreover, they can better articulate and provide a framework for analyzing the health effects of social interaction. Finally, the counterfactual approach made possible by agent-based modeling may promote causal thinking in social epidemiology and improve our mechanistic understanding and conceptual articulation of exposures when considering the social production of health.

However, several limitations need to be addressed as these approaches become more prevalent in social epidemiologic research. Considerable methodological development is needed in the area of longitudinal social network approaches to improve causal inference from social network analysis. Furthermore, social epidemiologists interested in agent-based approaches to etiologic inquiry need to develop "best practices" with regard ABM design, parameterization, interpretation, and validation in population health research.

## List of Abbreviations

ABM(s) is an abbreviation for agent-based models (or modeling). ABCs is an abbreviation for agent-based counterfactual simulations.

## Competing interests

The authors declare that they have no competing interests.

## Authors' contributions

AME conceived of the study and drafted the manuscript. PS advised on the manuscript design and critically revised the manuscript for intellectual content. LS helped with background research and critically revised the manuscript for intellectual content. SG advised on the manuscript design and critically revised the manuscript for intellectual content. All authors read and approved the final manuscript.
